# Kinetics of Arf1 inactivation regulates Golgi organisation and function in non-adherent fibroblasts

**DOI:** 10.1242/bio.059669

**Published:** 2023-05-04

**Authors:** Rajeshwari B.R., Nikita Shah, Prachi Joshi, M. S. Madhusudan, Nagaraj Balasubramanian

**Affiliations:** Indian Institute of Science Education and Research (IISER) Pune, Dr Homi Bhabha Road, Pashan, Pune, Maharashtra 411008, India

**Keywords:** Adhesion, Arf1, GBF1, Golgi, Kinetics

## Abstract

Arf1 belongs to the Arf family of small GTPases that localise at the Golgi and plasma membrane. Active Arf1 plays a crucial role in regulating Golgi organisation and function. In mouse fibroblasts, loss of adhesion triggers a consistent drop (∼50%) in Arf1 activation that causes the Golgi to disorganise but not fragment. In suspended cells, the trans-Golgi (GalTase) disperses more prominently than cis-Golgi (Man II), accompanied by increased active Arf1 (detected using GFP-ABD: ARHGAP10 Arf1 binding domain) associated with the cis-Golgi compartment. Re-adhesion restores Arf1 activation at the trans-Golgi as it reorganises. Arf1 activation at the Golgi is regulated by Arf1 Guanine nucleotide exchange factors (GEFs), GBF1, and BIG1/2. In non-adherent fibroblasts, the cis-medial Golgi provides a unique setting to test and understand the role GEF-mediated Arf1 activation has in regulating Golgi organisation. Labelled with Man II-GFP, non-adherent fibroblasts treated with increasing concentrations of Brefeldin-A (BFA) (which inhibits BIG1/2 and GBF1) or Golgicide A (GCA) (which inhibits GBF1 only) comparably decrease active Arf1 levels. They, however, cause a concentration-dependent increase in cis-medial Golgi fragmentation and fusion with the endoplasmic reticulum (ER). Using selected BFA and GCA concentrations, we find a change in the kinetics of Arf1 inactivation could mediate this by regulating cis-medial Golgi localisation of GBF1. On loss of adhesion, a ∼50% drop in Arf1 activation over 120 min causes the Golgi to disorganise. The kinetics of this drop, when altered by BFA or GCA treatment causes a similar decline in Arf1 activation but over 10 min. This causes the Golgi to now fragment which affects cell surface glycosylation and re-adherent cell spreading. Using non-adherent fibroblasts this study reveals the kinetics of Arf1 inactivation, with active Arf1 levels, to be vital for Golgi organisation and function.

## Teaser

**Summary:** This study uses Golgi disorganisation in non-adherent cells to test how differential Arf1 activation and kinetics of Arf1 inactivation could regulate Golgi organisation and function.

## INTRODUCTION

The Golgi apparatus is an intracellular membrane organelle that is seen to play a vital role in the trafficking and processing of proteins and lipids (Emr et al., 2009; Farquhar and Palade, 1998). Consisting of cis-, medial- and trans-cisternae, the Golgi stacks process and facilitate the targeting of newly synthesised cargo proteins as they emerge from the endoplasmic reticulum-Golgi intermediate compartment (ERGIC) and traffic through the trans-Golgi network. How individual compartments are created and maintained remains unclear, though recruiting protein enzymes and regulators to a specific compartment is vital to their establishment and function (Papanikou and Glick, 2014; Shorter and Warren, 2002). Among these regulators are Arf proteins, whose diverse functions include membrane trafficking, regulation of microtubules, and lysosome function (Donaldson and Jackson, 2011; Gillingham and Munro, 2007). There are three known classes of mammalian Arf proteins, Class I (Arfs1-3), Class II (Arfs 4-5), and Class III (Arf6) (Donaldson and Jackson, 2011; Manolea et al., 2010). Class I and II Arfs were found to be differentially distributed through the Golgi (Chun et al., 2008; Dejgaard et al., 2007; Manolea et al., 2010). Arf proteins exert their regulatory effect through cycles of guanosine-5'-triphosphate (GTP) binding and hydrolysis induced by Arf guanine-nucleotide-exchange factors (GEFs) and Arf GTPase-activating proteins (GAPs). Activation of Arf proteins occurs at the membrane and requires simultaneous membrane association of both substrate and the activating GEF (Cherfils and Melançon, 2005). The initial association of Arfs with membranes depends on its N-terminal myristoyl moiety (Franco et al., 1996; Haun et al., 1993; Randazzo and Kahn, 1995; Tsai et al., 1996). GTP loading induces the locking of the exposed N-terminal amphipathic motif of Arf1, allowing for its stable membrane association (Pasqualato et al., 2002). Arf GEFs release the guanosine diphosphate (GDP) bound to Arf1, which enables the GTP to bind. Arf GAPs drive the conversion of the Arf bound GTP to GDP, inactivating Arf1. This inactive Arf1 is displaced from the membrane, becoming more cytosolic (Bui et al., 2009). Studies have also suggested initial Arf association with membranes may depend on an Arf receptor that could be present on the Golgi membrane (Donaldson and Honda, 2005; Gommel et al., 2001).

At the Golgi, the most abundant Arf family member, Arf1 (Popoff et al., 2011), plays a vital role in the assembly and budding of coat protein (COPI) vesicles (Bremser et al., 1999; Kahn and Gilman, 1984; Ostermann et al., 1993). Arf1 has emerged as a master regulator of the Golgi function (Jackson and Bouvet, 2014; Pasqualato et al., 2002). Arf1-GDP is recruited to the cis-Golgi membrane by p24 family proteins (Gommel et al., 2001), which was activated by the GEF GBF1 (Claude et al., 1999). Active ARF1 then recruits coatomer (Palmer et al., 1993; Serafini et al., 1991), driving the formation of COPI-coated vesicles at the cis-Golgi. Arf1 also helps recruit lipid-modifying enzymes that regulate the lipid composition of the Golgi membrane, helping differentiate it from the ER membrane (Kaczmarek et al., 2017; Memon, 2004; Presley et al., 2002; Spang, 2002). In addition to its role in COPI-mediated retrograde and intra-Golgi transport, Arf1 has also been shown to recruit the adaptor protein complexes AP1, AP3, and AP4, as well as Golgi-localizing, gamma-adaptin ear homology domain, ARF-binding protein (GGA) complexes (Boehm et al., 2001; Bonifacino, 2004; Ooi et al., 1998; Stamnes and Rothman, 1993; Traub et al., 1993) and exomer complexes (Paczkowski and Fromme, 2014) at the trans-Golgi network, controlling vesicle formation and transport (D'Souza-Schorey and Chavrier, 2006; Nie et al., 2003; Ren et al., 2013). Arf1 is also involved in regulating the organisation of the Golgi membrane at the microtubule-organizing centre (MTOC) by mediating the association of the Golgi membrane with the microtubules (Thyberg and Moskalewski, 1999; Yadav et al., 2012).

Inactivation of Arf1 by drugs or mutation causes the disassembly of the Golgi apparatus and disrupts Golgi-dependent trafficking pathways (Klausner et al., 1992; Sáenz et al., 2009). Brefeldin A (BFA) (Doms et al., 1989; Fujiwara et al., 1988; Lippincott-Schwartz et al., 1989) acts as an uncompetitive inhibitor of a sub-family of large ArfGEFs that includes Golgi-specific Brefeldin A-resistance factor 1 (GBF1) and BFA-inhibited GEFs (BIG1/2) (Casanova, 2007). BIG1/2 and GBF1, like other members of the Sec7 family of Adenosine diphosphate (ADP, also known as adenosine pyrophosphate) ribosylation factor (Arf) guanine nucleotide-exchange factors (GEFs), drive the replacement of Arf-bound GDP with GTP to generate active Arf-GTP (Bui et al., 2009; Claude et al., 1999; Jackson, 2018; Manolea et al., 2008). GBF1 and BIG1/2 also share conserved homology domains (Bui et al., 2009; Mouratou et al., 2005), which could modulate the location and extent of Arf1 activation. BIG1 was seen to localise at the trans-Golgi network, partially overlapping with BIG2 (Manolea et al., 2008; Boal and Stephens, 2010; Yamaji et al., 2000). Their functions at the trans-Golgi network, while redundant (Ishizaki et al., 2008), BIG1 and BIG2 were also thought to have unique roles. The activation of Arf1 at the cis-Golgi is regulated by GBF1 (Kawamoto et al., 2002; Lowery et al., 2013; Manolea et al., 2008). Golgicide A (GCA) was seen to act specifically on GBF1 to inactivate Arf1 and regulate Golgi organisation (Sáenz et al., 2009). The organisation of the Golgi membranes plays a vital role in regulating cargo processing and trafficking (Glick, 2000). It influences the spatial separation of glycosylation enzymes providing a suitable environment for enzyme activity. The stacking of Golgi membranes limits the rate of cargo movement ensuring most cargo sorting happens at the trans-Golgi (Lowe, 2011). Changes in the Golgi organisation influence other cellular events like cell division, migration, and signalling in cells (Hicks and Machamer, 2005; Xing et al., 2016; Yadav et al., 2009).

Earlier studies from the lab have shown that cell-matrix adhesion can regulate Arf1 activation at the Golgi, which controls Golgi organisation and function (Singh et al., 2018). Loss of adhesion led to a ∼50-60% drop in Arf1 activity in wild-type mouse embryonic fibroblasts (WT-MEFs), causing the Golgi to disorganise distinctly different from known Golgi fragmentation (Lippincott-Schwartz et al., 1989; Singh et al., 2018). This, when considered with the fact that the trans-Golgi in non-adherent fibroblasts is distinctly more disorganised than the cis-Golgi, suggests a differential role for adhesion-dependent Arf1 activation across Golgi compartments. Further, in non-adherent cells, a drop in active Arf1 levels caused by the inhibition of Arf GEFs (BFA/GCA treatment) drives Golgi fragmentation (Singh et al., 2018). Non-adherent mouse fibroblasts provide a unique scenario to evaluate and understand the regulation of Arf1 inactivation and its role in Golgi organisation. Whether net active Arf1 levels alone determine how the Golgi is organised (in stable adherent), disorganised (in suspension), or fragmented (suspension+GEF inhibition) remains a question of interest. Using Arf GEF inhibitors (BFA/GCA) to titrate active Arf1 levels in suspended and early detached cells helps reveal the kinetics of Arf1 inactivation to be vital to regulating Golgi organisation and function.

## RESULTS

### Adhesion-dependent differential Arf1 activation regulates cis- versus trans-Golgi organisation

**Fig. 1.**
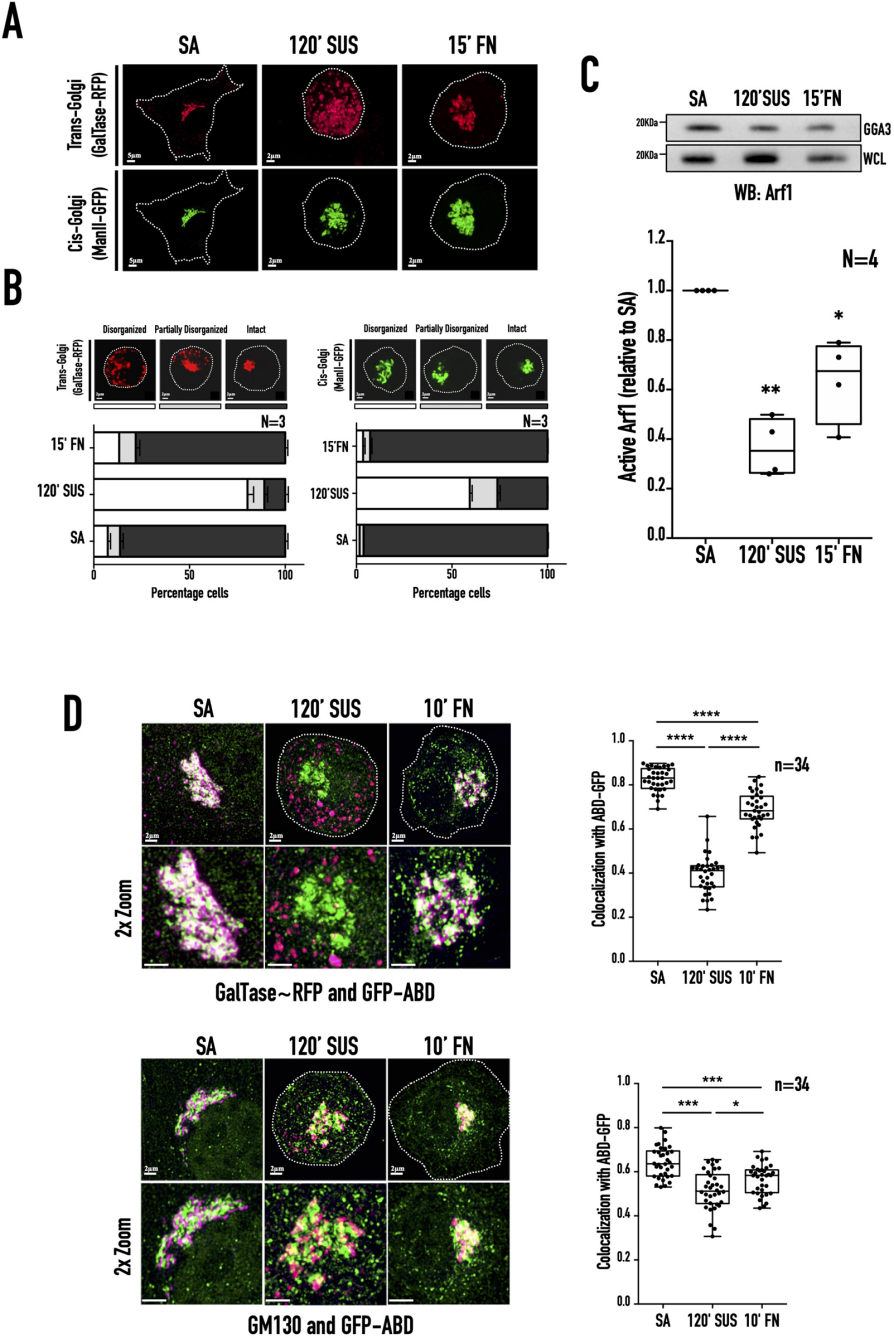
**Adhesion-dependent Arf1 activation differentially regulates cis-medial- versus trans-Golgi organisation in WT-MEFs.** (A) Representative de-convoluted maximum intensity projection (MIP) images of the Golgi in WT-MEFs transfected with GalTase-RFP (trans-Golgi marker) and ManII-GFP (cis-Golgi marker) when stable adherent (SA), held in suspension for 120 min (120′ SUS), and re-adherent on FN for 15 min (15′ FN). (B) The percentage distribution profile of WT-MEFs with disorganised (clear), partially disorganised (grey), and intact (black) cis-medial Golgi (ManII-GFP) and trans-Golgi (GalTase-RFP) was calculated. The graph represents their mean±standard error (SE) of percentage distribution in SA, 120′ SUS, and 15′FN from three independent experiments. (C) Western blot detection of active Arf1 (WB: Arf1) pulled down using GST-GGA3 (GGA3 PD) and total Arf1 in the whole-cell lysate (WCL) from SA, suspended (120′ SUS), and re-adherent (15′ FN) WT-MEFs. The box and whisker plot represents the densitometric band intensity ratio of active to total Arf1 from four independent experiments, normalised to stable adherent cells. (D) Representative de-convoluted MIP images of SA, suspended (120′ SUS), and re-adherent (15′ FN) WT-MEFs expressing GFP-ABD (shown in green) with GalTase-RFP (shown in magenta) (top panel) or immunostained with GM130 (shown in magenta) (lower panel). The box and whisker plot represents Pearson's coefficient of co-localisation for GFP-ABD with GalTase-RFP or GM130 in 34 cells from three independent experiments. Statistical analysis was using the Mann–Whitney test, *P<0.05, **P<0.001, ***P<0.0001, ****P<0.00001, or with normalised data using single sample *t*-test, *P<0.05, **P<0.001, ***P<0.0001, ****P<0.00001.

Cell-matrix adhesion-dependent Arf1 activation regulates Golgi organisation in MEFs (Singh et al., 2018). It causes the Golgi to distinctly disorganise on the loss of adhesion and rapidly reorganise on re-adhesion. In serum-deprived stable adherent WT-MEFs, the cis-medial Golgi marker Mannosidase II–GFP and trans-Golgi marker GalTase-RFP show an almost complete overlap in the organised Golgi (Fig. 1A). When cells were detached and held in suspension for 120 min (120′ SUS), the cis-medial and trans-Golgi were seen to disorganise differently (Fig. 1A). The trans-Golgi was extensively dispersed, occupying most of the cell volume. At the same time, the cis-Golgi is less dispersed and stays largely perinuclear (Fig. 1A). This is the predominant phenotype observed and confirmed by their distribution profile in suspended cell populations (Fig. 1B). Upon re-adhesion on fibronectin for 15 min (15′ fibronectin (FN), both Golgi compartments rapidly reorganise (Fig. 1A) around the MTOC (Singh et al., 2018), confirmed by their distribution profile (Fig. 1B). We further tested and confirmed the adhesion-dependent regulation of Arf1. Loss of adhesion caused a significant decrease in Arf1 activation that was restored on re-adhesion (Fig. 1C). This represents the total cellular Arf1 activation status in cells and leads to the speculation that differential activation of Arf1 in the cis- versus trans-Golgi could drive their differential disorganisation on the loss of adhesion. To test this, we expressed the GFP-ABD construct known to bind active Arf1 and evaluated its localisation with cis- (GM130) and trans-Golgi (GalTase) compartments in non-adherent WT-MEFs. Colocalisation of GFP-ABD with GalTase-RFP (Trans-Golgi) in stable adherent cells drops significantly in suspended cells (where the trans-Golgi was extensively dispersed) and recovers on re-adhesion (Fig. 1D). In contrast, co-localisation of green fluorescent protein-Arf binding domain (GFP-ABD) with GM130 (cis-Golgi) drops only marginally in suspended cells and recovers on re-adhesion (Fig. 1D). This suggests on the loss of adhesion much of the decrease in net Arf1 activation (Fig. 1C) was at the trans-Golgi and not the cis-Golgi compartment. Arf1 GEF, GBF1 is known to localise to cis-Golgi while BIG1/2 localises to the trans-Golgi in cells (Kawamoto et al., 2002; Lowery et al., 2013; Manolea et al., 2008). The differential localisation and regulation of Arf GEFs in the trans- versus cis-Golgi, could help mediate differences in Arf1 activation between these compartments.

### Relative expression of Arf1-GEFs and their targeting with inhibitors

**Fig. 2.**
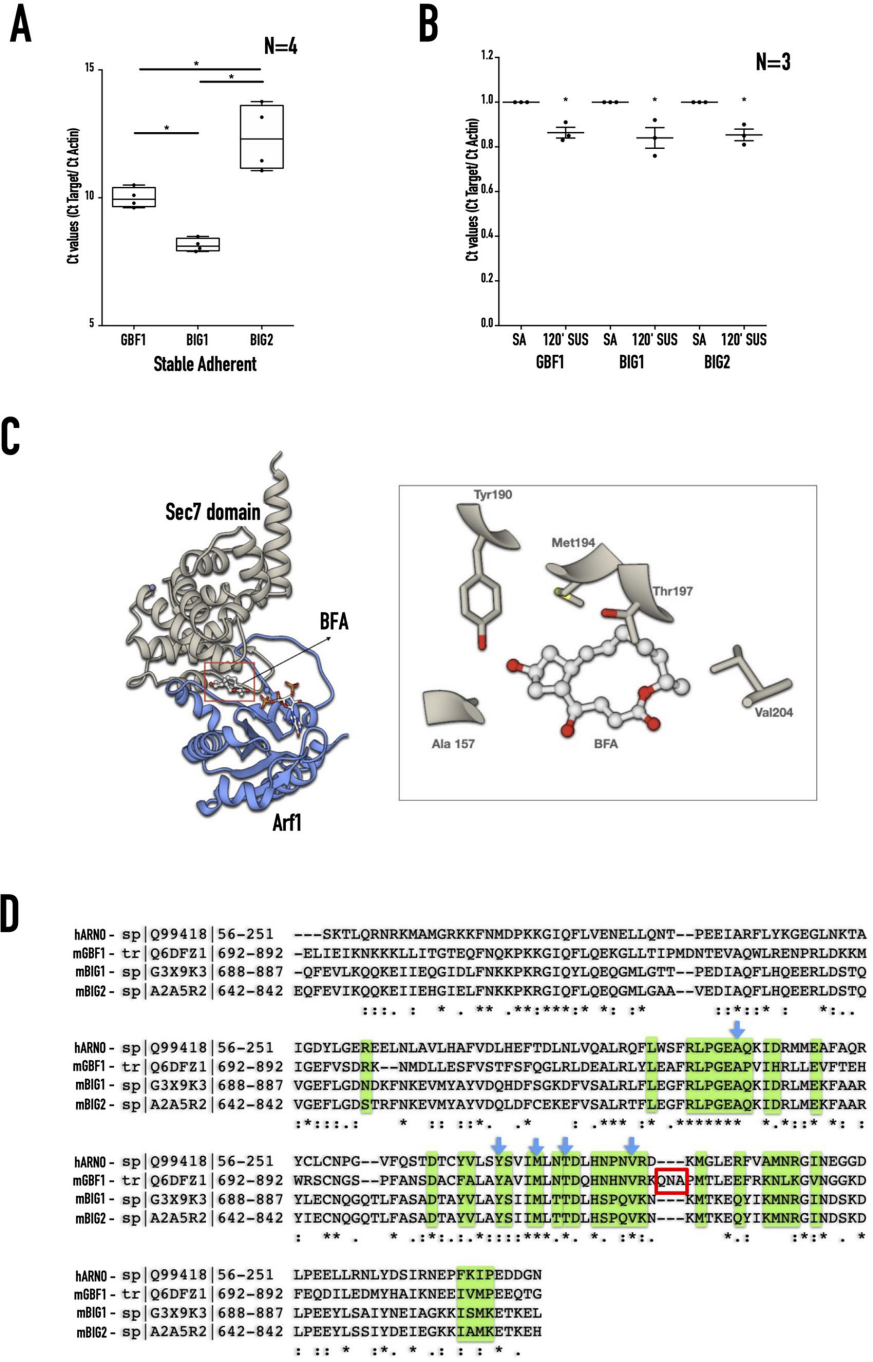
**Arf1-GEFs expression in WT-MEFs.** (A) Arf GEF expression profile in stable adherent WT-MEFs detected by RT-PCR shows BIG1 to be better expressed (lower Δ Ct values) than GBF1 and BIG2. The box and whisker plot represents the Δ Ct values relative to actin from four independent reactions. Statistical analysis of normalised data was done using the single-sample *t*-test (*P<0.05). (B) Arf GEF expression profile in WT-MEFs detected by RT-PCR compares the expression of GBF1, BIG1, BIG2 in SA and suspended (120′ SUS) cells. The box and whisker plot represents the Δ Ct values for each GEF in 120′ SUS cells relative to SA cells relative to respective Δ Ct values of actin from four independent reactions. All data were analysed using the single sample t-Test, and *P* values are as indicated (* *P*<0.05). (C) Ribbon representation of the Arf-GEF Sec7 domain in complex with BFA. Only the BIG1 Sec7-BFA-Arf1 complex is represented here. The sec7 domain of BIG1 is in blue ribbons, and Arf1 is represented in grey ribbons. The BFA sandwiched between the Arf1 and GEF is shown in a ball and stick representation. The inset shows the residues marked by blue arrows in Fig. 2D represented as sticks interacting with BFA (in a ball and stick representation). The inset was rendered using UCSF Chimera (Pettersen et al., 2004). (D) Multiple sequence alignment of the sec7 domains of the mouse BIG1, BIG2, GBF1, and human ARNO as constructed by Clustal Omega. The amino acids highlighted in green interact with Arf1 (within 4.0 Å) in the homology models. The residue positions marked with blue arrows are the residues making contact (within 4.0 Å) with BFA in these models. The red box marks the QNA residues unique to GBF1 and responsible for its specific interactions with GCA (Sáenz et al., 2009).

As a first step to determine their relative role, we tested the relative expression of Arf1 GEFs, GBF1, and BIG1/BIG2 in adherent versus non-adherent WT-MEFs by qRT-PCR. It identified BIG1 expression to be the highest, followed by GBF1 and BIG2 in serum-deprived stable adherent WT-MEFs (Fig. 2A) (BIG1>GBF1>BIG2). All three GEFs showed a ∼15% drop in their mRNA levels in suspended WT-MEFs (Fig. 2B) which could, in turn, affect their protein levels. However, their relative expression in non-adherent cells is comparable to stable adherent cells (BIG1>GBF1>BIG2). Therefore, targeting these GEFs using inhibitors BFA and GCA could help evaluate their relative role in regulating Arf1 activation and function. BFA is an uncompetitive inhibitor of a sub-family of ArfGEFs, including GBF1 and BIG1/BIG2 (BFA-inhibited GEFs) (Casanova, 2007). However, GCA inhibits GBF1 without affecting BIG1/BIG2 (Sáenz et al., 2009). BFA is known to bind a highly conserved Sec7 domain in Arf GEFs to inhibit Arf1 GDP-GTP exchange (Niu et al., 2005, Peyroche et al., 1999). Using the known human Arf nucleotide binding site opener (ARNO) Sec7-BFA-Arf1 structure (PDB ID:1R8Q), three-dimensional models were generated for the Sec7 domain of mouse BIG1, BIG2, and GBF1 in a complex with BFA and mouse Arf1 (representative image for BIG1-BFA-Arf1 shown in Fig. 2C). It reveals residues vital for GEF interaction with BFA (right panel Fig. 2C) were conserved between human (ARNO) and mouse Arf1 GEFs (BIG1, BIG2, and GBF1) (Fig. 2D marked by blue arrows). It suggests that the inhibitory effect of BFA should be comparable across these GEFs. GCA also binds the same Sec7 pocket, its interaction extending beyond to contact the tripeptide loop made by the Glutamine, Aspergine, Alanine (QNA) residues in GBF1 (Fig. 2C, highlighted with a red box), which allows it to inhibit GBF1 specifically (Sáenz et al., 2009).

### BFA-mediated decrease in Arf1 activation regulates Golgi fragmentation in non-adherent WT-MEFs

**Fig. 3.**
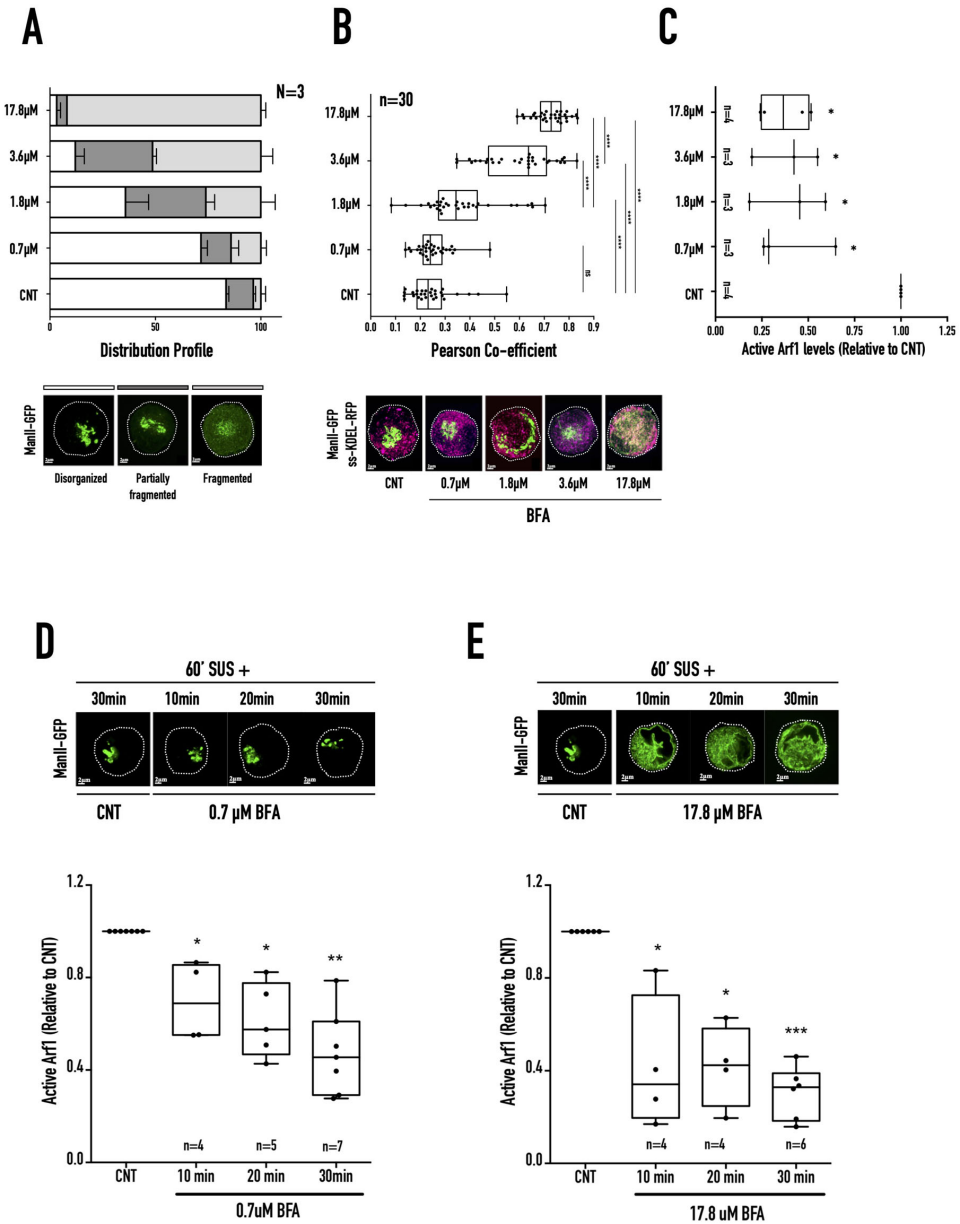
**Concentration-dependent BFA treatment of suspended WT-MEFs.** (A) WT-MEFs expressing cis-medial Golgi (ManII-GFP) marker were held in suspension for 60 min and treated for 30 min without (CNT) or with increasing concentrations of BFA (0.7 µM, 1.8 µM, 3.6 µM, 17.8 µM). The distribution of cells with disorganised (clear), partially fragmented (dark grey), and fragmented (grey) Golgi were manually counted, and the graph represents their mean±SE of percentage distribution from three independent experiments. Selected representative images for each phenotype are presented below the graph. (B) The box and whisker plot represent the Pearson's coefficient of co-localisation for cis-Golgi (ManII-GFP) and ER (ss-KDEL-RFP) in WT-MEFs suspended for 60 min and treated for 30 min without (CNT) or with BFA (0.7 µM, 1.8 µM, 3.6 µM, 17.8 µM), 30 cells from three independent experiments. Selected representative images for the prominent phenotype of each BFA concentration are presented below the graph, ManII-GFP is shown in green, and ss-KDEL-RFP is shown in magenta. (C) Western-blot detection of active Arf1 pulled down using GST-GGA3 and equated to total Arf1 in whole-cell lysate from WT-MEFs in suspension for 60 min and treated for 30 min without CNT or with BFA (0.7 µM, 1.8 µM, 3.6 µM, or 17.8 µM). The box and whisker plot represents the densitometric band intensity ratio of active to total Arf1 normalised to respective control (CNT) from three independent experiments. (D,E) Representative images of cis-medial Golgi (ManII-GFP) phenotype in cells held in suspension for 60 min (60′ SUS+), treated without (CNT) or with (D) low BFA **(**0.7 µM) or (E) high BFA (17.8 µM) for 10 min, 20 min, and 30 min, respectively. The box and whisker plot represents the densitometric band intensity ratio of active to total Arf1 normalised to respective control (CNT) from three to six independent experiments. Statistical analysis was done using the Mann–Whitney test, *P<0.05, **P<0.001, ***P<0.0001, ****P<0.00001, or with normalised data using single sample *t*-test, *P<0.05, **P<0.001, ***P<0.0001, ****P<0.00001.

Non-adherent WT-MEFs with a disorganised Golgi provide a unique cellular setting to study Arf1 activation-mediated regulation of Golgi organisation. BFA, as an uncompetitive inhibitor of GBF1 and BIG1/2 (Casanova, 2007), inhibits Arf1 to cause the cis/cis-medial Golgi to fragment and fall back into the ER in stable adherent (Klausner et al., 1992) and non-adherent cells (Singh et al., 2018). In suspended cells, this could be mediated by the targeting of GBF1 at the cis/cis-medial Golgi to affect Arf1 activation (Fig. 1). WT-MEFs expressing the cis-medial Golgi marker ManII-GFP were suspended for 60 min and treated with increasing BFA concentrations (0.7 µM, 1.8 µM, 3.6 µM, 17.8 µM) for 30 min. The disorganised Golgi (ManII-GFP) in suspended cells show a concentration-dependent increase in its fragmentation (Fig. 3A). This is reflected in a simultaneous increase in its co-localisation with the ER lumen marker ss-RFP-KDEL (Fig. 3B) (Altan-bonnet et al., 2006). ss-RFP-KDEL localisation is not affected by BFA treatment (Fig. S1C). The BFA concentration-dependent Golgi fragmentation could result from BFA-mediated differential inhibition of Arf1 activation. Interestingly, when tested, Arf1 activation was seen to drop by ∼50-60% relative to control across all BFA concentrations (Fig. 3C) (Fig. S1A). However, there was no significant change in the total Arf1 levels across all treatments (Fig. S1B). This suggests that the net active Arf1 levels in non-adherent fibroblasts alone might not be enough to regulate Golgi fragmentation. The kinetics of Arf1 inactivation on treatment with increasing BFA concentration could drive Golgi fragmentation. Treatment with 0.7 µM and 17.8 µM BFA results in a comparable decrease in net active Arf1 levels with distinct differences observed in Golgi fragmentation (Fig. 3B,C). We chose these concentrations to test their effect on the kinetics of Arf1 inactivation on 30 min of treatment. With 0.7 µM BFA treatment, Arf1 activity drops gradually by ∼30% (10 min), ∼39% (20 min), and ∼53% (30 min) over time (Fig. 3D; Fig. S1B,S1D). In comparison, 17.8 µM BFA causes a ∼58% drop in Arf1 activation within the first 10 min that was retained at 20 min (∼59% decrease) and 30 min (∼70% decrease) (Fig. 3E) (Fig. S1C,S1D). There was no significant change in total Arf1 levels when cells were treated with 0.7 µM or 17.8 µM BFA for increasing times (Fig. S1F). Treatment with 0.7 µM BFA caused a gradual drop in Arf1 activation by ∼53% over 30 min but did not cause fragmentation of ManII-GFP labelled Golgi (Fig. 3D). In contrast, 17.8 µM BFA caused a steep drop in Arf1 activation by ∼58% in 10 min, caused the Golgi to fragment (Fig. 3E). This suggests BFA-mediated inhibition of Arf GEFs and the resulting kinetics of change in Arf1 activation could drive Golgi organisation in non-adherent WT-MEFs.

### GCA-mediated decrease in Arf1 activation regulates Golgi fragmentation in non-adherent WT-MEFs

**Fig. 4.**
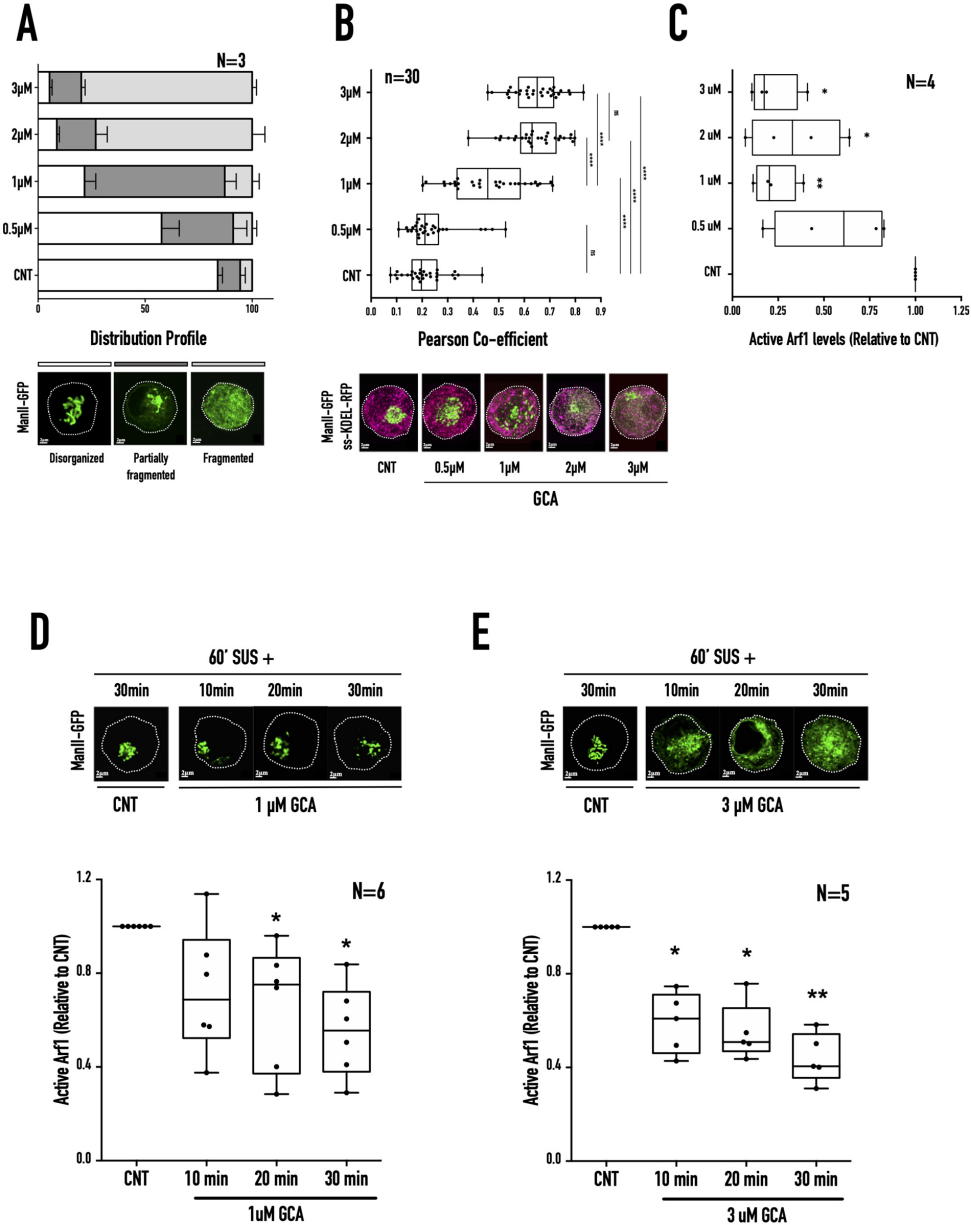
**Concentration-dependent GCA treatment of suspended WT-MEFs.** (A) WT-MEFs expressing cis-medial Golgi (ManII-GFP) marker were held in suspension for 60 min treated for 30 min without (CNT) or with increasing concentrations of GCA (0.5 µM, 1 µM, 2 µM, 3 µM). The distribution of cells with disorganised (clear), partially fragmented (dark grey), and fragmented (grey) Golgi were manually counted, and the graph represents their mean±SE of percentage distribution from three independent experiments. Selected representative images are presented below the graph. (B) The box and whisker plot represent the Pearson's coefficient of co-localisation for cis-medial Golgi (ManII-GFP) and ER (ss-KDEL-RFP) in WT-MEFs suspended for 60 min and treated for 30 min without (CNT) or with GCA (0.5 µM, 1 µM, 2 µM, 3 µM), 30 cells from three independent experiments. Selected representative images for the prominent phenotype of each GCA concentration are presented below the graph, ManII-GFP is shown in green, and ss-KDEL-RFP is shown in magenta. (C) Western-blot detection of active Arf1 pulled down using GST-GGA3 and equated to total Arf1 in whole-cell lysate from WT-MEFs suspended for 60 min and treated for 30 min without (CNT) or with GCA (0.5 µM, 1 µM, 2 µM, 3 µM). The box and whisker plot represents the densitometric band intensity ratio of active to total Arf1 normalised to respective control (CNT) from three independent experiments. (D,E) Representative images of cis-medial Golgi (ManII-GFP) phenotype in cells held in suspension for 60 min (60′ SUS+), treated without (CNT) or with (D) low GCA (1 µM) or (E) high GCA (3 µM) for 10 min, 20 min, and 30 min. The box and whisker plot represents the densitometric band intensity ratio of active to total Arf1 from five or six independent experiments. Statistical analysis was using the Mann–Whitney test, *P<0.05, **P<0.001, ***P<0.0001, ****P<0.00001, or normalised data using single sample *t*-test, *P<0.05, **P<0.001, ***P<0.0001, ****P<0.00001.

**Fig. 5.**
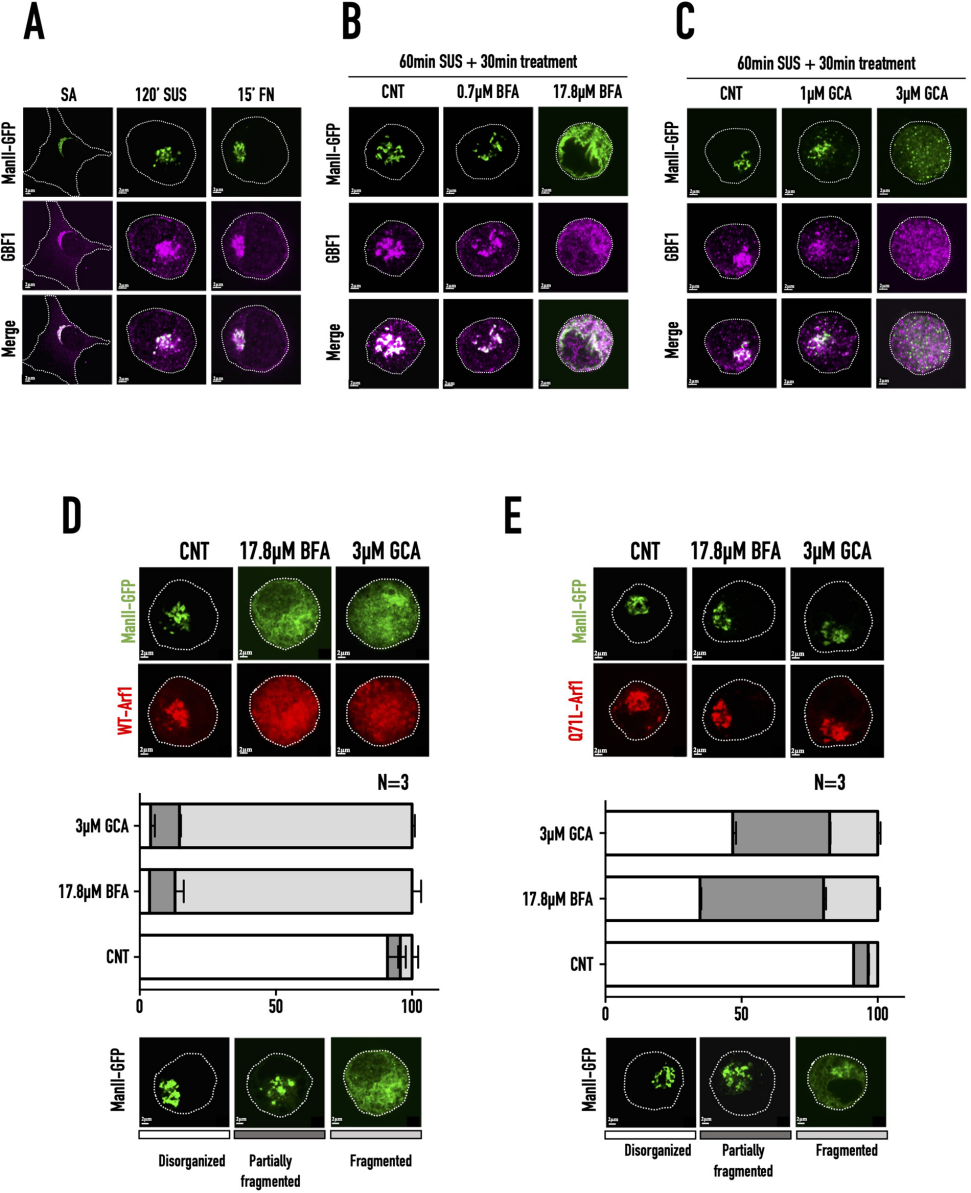
**Localisation of GBF1 and ManII-GFP labelled Golgi in WT-MEFs.** (A) Representative immunostained images of GBF1 in ManII-GFP expressing WT-MEFs, SA, suspended (120′ SUS), and re-adherent on fibronectin (15′ FN). (B,C) ManII-GFP expressing WT-MEFs (CNT) suspended for 60 min and treated with (B) 0.7 µM BFA or 17.8 µM BFA for 30 min (60′ SUS+30 min treatment) or (C) 1 µM GCA or 3 µM GCA for 30 min (60′ SUS+30 min treatment) and immunostained with GBF1. Representative ManII-GFP, GBF1, and merged images are shown. (D,E) WT-MEFs expressing (D) WT-Arf1-mCherry or (E) Q71L-Arf1-mCherry held in suspension for 60 min and treated with 17.8 µM BFA or 3 µM GCA for30 min (60′ SUS+30 min treatment). The upper panel shows representative images of the predominant Golgi phenotype (ManII-GFP) in (D) WT-Arf1-mCherry or (E) Q71L-Arf1-mCherry expressing cells. The graph shows the percentage distribution of cells with disorganised (clear), partially fragmented (dark grey), and fragmented (grey) Golgi, represented as their mean±SE from three independent experiments. Selected representative images for each Golgi phenotype (ManII-GFP) are presented below the graph.

GBF1 is the primary GEF responsible for the activation of Arf1 at the ERGIC and cis-Golgi membranes (Kawamoto et al., 2002; Manolea et al., 2008; Zhao et al., 2002; 2006). BFA treatment's effect on the cis-medial Golgi in non-adherent cells could reflect its regulation of GBF1. To confirm this, we tested the impact GBF1-specific inhibitor GCA (Sáenz et al., 2009) has on Arf1 activation and Golgi fragmentation in suspended cells. WT-MEFs transfected with ManII-GFP suspended for 60 min were treated with increasing concentrations of GCA (0.5 µM, 1 µM, 2 µM, 3 µM) for 30 min. The disorganised Golgi (ManII-GFP) showed a concentration-dependent increase in its fragmentation (Fig. 4A). This reflects in its increased co-localisation with the ER marker ss-RFP-KDEL (Fig. 4B). ss-RFP-KDEL distribution is unaffected by GCA treatment (Fig. S2C). The higher 2 µM and 3 µM GCA concentrations had comparable effects on the Golgi distribution profile and ER fallback. We further tested the effect of GCA treatment on active Arf1 levels. We found active Arf1 levels reduced comparably (by ∼65-75%) relative to control across GCA concentrations (Fig. 4C) (Fig. S2A), except for 0.5 µM GCA (∼45% decrease) (Fig. 4C). There was no significant change in the total Arf1 levels across all treatments (Fig. S2B). This is comparable to how increasing BFA concentrations affect Golgi organisation and Arf1 activation (Fig. 3B,C) supporting the role kinetics of drop in Arf1 activation could have in mediating the same. Treatment with 1 µM and 3uM GCA shows a comparable decrease in net active Arf1 levels but a distinct difference in their Golgi fragmentation profile (Fig. 4B,C). To test their effect on the kinetics of Arf1 inactivation, WT-MEFs suspended for 60 min were incubated with GCA (1 µM and 3 µM) for 10 min, 20 min, and 30 min, respectively, and their Arf1 activation was compared. 1 µM GCA treatment caused Arf1 activity to drop by ∼28% (10 min), ∼34% (20 min), and ∼45% (30 min) (Fig. 4D) (Fig. S2B, S2D). In comparison, 3 µM GCA treatment causes a ∼41% drop in Arf1 activation within the first 10 min that decreases by ∼45% at 20 min, and ∼56% at 30 min (Fig. 4E) (Fig. S2C, S2D). No significant change in total Arf1 levels was observed across these treatments (Fig. S2F). Treatment with 1 µM GCA caused a gradual drop in Arf1 activation by ∼45% over 30 min. Still, it did not cause fragmentation of ManII-GFP labelled Golgi (Fig. 4D). In contrast, 3 µM GCA caused a steep drop in Arf1 activation by ∼41% in 10 min, caused the Golgi to fragment (Fig. 4E). This could be mediated by their inhibition of GBF1 that stays localised with cis-medial Golgi (ManII-GFP) in suspended cells, comparable to stable adherent and re-adherent cells (Fig. 5A). Treatment with 0.7 µM BFA or 1 µM GCA, which did not cause Golgi fragmentation, did not displace GBF1 from the cis-medial Golgi, unlike when treated with 17.8 µM BFA or 3 µM GCA (Fig. 5B and C). This suggests that BFA and GCA-mediated regulation of GBF1 could drive the differential kinetics of change in Arf1 activation to affect cis-medial Golgi fragmentation.

### Constitutive active Arf1 (Q71L) rescues BFA/GCA mediated Golgi fragmentation

To confirm the role regulation of Arf1 activation has in driving BFA or GCA-mediated Golgi fragmentation, we expressed constitutively active Q71L-Arf1-mCherry (or WT-Arf1-mCherry) with ManII-GFP in WT-MEFs. These cells held in suspension for 60′ were treated with 17.8 µM BFA or 3 µM GCA for 30 min, the highest concentration of BFA or GCA seen to completely fragment the Golgi in earlier experiments (Figs 3A and 4A). Furthermore, the effect that constitutively active Arf1 (Q71L) or WT-Arf1 has on Golgi organisation in these inhibitor-treated cells was compared. In WT-Arf1 expressing cells treated with BFA or GCA, ∼87% and ∼85% of cells showed Golgi fragmentation (Fig. 5D). Constitutively active Arf1 (Q71L) expression caused their numbers to drop to ∼19% and ∼17% respectively (Fig. 5E). An increase in the percentage of cells with disorganised Golgi was also observed in Q71L-Arf1 expressing cells treated with BFA (∼35%) or GCA (∼46%). This is a distinctly different in WT Arf1-expressing cells treated with BFA (–3.6%) or GCA –3.9%). Together this reveals that constitutively active Arf1 can rescue Arf GEF inhibitor- (BFA/GCA) mediated fragmentation of Golgi in suspended WT-MEFs.

### Kinetics of Arf1 inactivation regulates loss of adhesion-mediated Golgi disorganisation

**Fig. 6.**
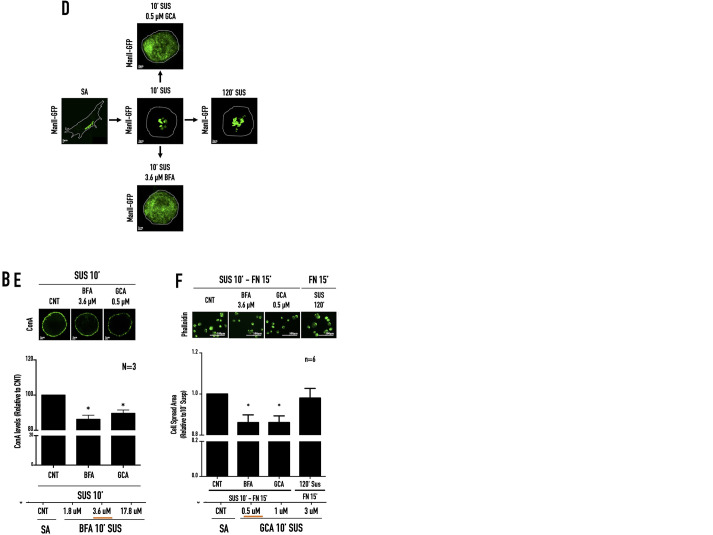
**Kinetics of Arf1 inactivation on the loss of adhesion in WT-MEFs.** Western-blot detection of active Arf1 (WB: Arf1) pulled down using GST-GGA3 (GGA3 PD) and total Arf1 in the whole-cell lysate (WCL) from WT-MEFs, (A) detached and held in suspension for increasing time (10′, 30′, 60′, 90′, 120′ SUS) relative to SA cells or (B,C) cells suspended and treated for 10 min in suspension, with (B) BFA 1.8 µM, 3.6 µM and 17.8 µM, or (C) GCA 0.5 µM, 1 µM, and 3 µM relative to respective stable adherent control cells (CNT, untreated). The box and whisker plot represents the densitometric band intensity ratio of active to total Arf1 normalised to respective control (CNT) from three independent experiments. (D) Representative cross-section images of cis-Golgi (ManII-GFP) phenotype in WT-MEFs, SA, suspended for 10 min (10′ SUS), 120 min (120′ SUS), or suspended for 10 min (10′ SUSP) with 0.5 µM GCA and 3.6 µM BFA. (E) WT-MEFs held in suspension for 10 min (SUS 10′), without (CNT), or with 3.6 µM BFA or 0.5 µM GCA was surface labelled with ConA-Alexa-488. The graph represents the median surface-bound ConA fluorescence normalised to CNT from three independent experiments. Representative images are shown above the graph. (F) WT-MEFs held in suspension for 10 min (SUS 10′), without (CNT), or with 3.6 µM BFA, or 0.5 µM GCA or in suspension for 120 min, were replated on fibronectin for 15 min (FN15′) and cell spreading was evaluated by phalloidin staining. The box and whisker plot represents six independent experiments’ mean cell-spread area. Representative images are shown above their respective graph. Statistical analysis was using the Mann–Whitney test, *P<0.05, **P<0.001, ***P<0.0001, ****P<0.00001, or for normalised data using single sample *t*-test, *P<0.05, **P<0.001, ***P<0.0001, ****P<0.00001.

The above inhibitor studies (BFA/GCA) suggest that the kinetics of change in active Arf1 levels is vital for regulating Golgi organisation. The physiological loss of adhesion-mediated Golgi disorganisation in being dependent on Arf1 (Fig. 1C, Singh et al., 2018) could be similarly regulated by the kinetics of change in active Arf1 levels. WT-MEFs were suspended for 10, 30, 60, 90, and 120 min, and active Arf1 levels were determined, which showed a steady decrease in active Arf1 levels over time. A 15-20% drop in Arf1 activation in 10 and 30 min, drops by ∼33% in 60 min, ∼40% in 90 min, and eventually ∼53% in 120 min, relative to stable adherent cells (Fig. 6A). The kinetics of this change in active Arf1 levels could be vital to the Golgi being distinctly disorganised on loss of adhesion. To test this, we asked whether disrupting these kinetics without significantly affecting the net active Arf1 levels can affect Golgi disorganisation on loss of adhesion. Arf1 activity drops by ∼50% in WT-MEFs suspended for 120 min. Therefore, we used a range of BFA and GCA concentrations to determine the lowest concentration of each inhibitor that causes a similar ∼50% drop in Arf1 activity, but in 10 min of treatment of detached cells. This revealed 3.6 µM BFA and 0.5 µM GCA treatment to cause a ∼54% and ∼45% drop in Arf1 activity in 10 min in detached WT-MEFs (Fig. 6B and C, respectively). The treatment of detached cells with intact Golgi that is becoming disorganised makes the kinetics of change in Golgi organisation observed on BFA/GCA treatment marginally different from that observed in suspended cells (for 60 min) treated with BFA/GCA (Figs 3C and 4C). BFA (3.6 µM)- or GCA (0.5 µM)-mediated Arf1 inactivation in detached cells causes the Golgi (Man II GFP) to fragment, unlike on loss of adhesion (for 120 min) where it disorganises (Fig. 6D; Fig. S3). Both have comparable net active Arf1 levels (Fig. 6A,B,C) but vary in their kinetics of drop in active Arf1 levels. Golgi organisation affects the trafficking and processing of proteins (Pokrovskaya et al., 2011). Loss-of-adhesion-mediated disorganisation of the Golgi affects cell-surface glycosylation (Singh et al., 2018), detected by lectin binding. WT-MEFs suspended for 10 min with 3.6 µM BFA, or 0.5 µM GCA showed a significant reduction in cell surface ConA binding relative to control cells (Fig. 6E). Also, early adhesion-dependent spreading of 3.6 µM BFA- or 0.5 µM GCA-treated detached cells were significantly reduced as compared to control (10 min SUS) and suspended cells (120 min SUS) (Fig. 6F). Together they reveal that the kinetics of Arf1 activation in cells controls Golgi organisation and function.

## DISCUSSION

Working with Golgi matrix proteins and cytoskeleton components, the small GTPase Arf1 is a vital regulator of Golgi organisation and function downstream of multiple stimuli. In mouse fibroblasts, loss of adhesion is accompanied by a consistent drop in Arf1 activation, causing the Golgi to disorganise but not to fragment (Fig. 1A,B) (Altan-Bonnet et al., 2006; Lippincott-Schwartz et al., 1989; Singh et al., 2018). The distinct regulation of the Golgi on loss of adhesion was further illustrated by the differential disorganisation of the trans-Golgi (GalTase) and cis-medial Golgi (Man II) (Fig. 1B). This could be mediated by the differential activation of Arf1 (detected using GFP-ABD) in these compartments (Fig. 1D). On loss of adhesion the trans-Golgi loses active Arf1, unlike the cis-medial Golgi. Active Arf1 retained at the cis-medial Golgi (Fig. 1D) allows us to test further its role and regulation in the Golgi organisation in suspended cells. Loss-of-adhesion-dependent signalling in suspended cells means this regulation of the Golgi will be without interference from adhesion-dependent regulatory pathways. This unique circumstance allows us to test the Arf1-dependent regulation of the Golgi in a way that has not been done before. Arf1 localisation at the Golgi depends on its activation by Arf-GEFs GBF1 and BIG1/2 (Manolea et al., 2008). Their relative expression in WT-MEFs (BIG1> GBF1> BIG2) is unaffected by loss of adhesion (Fig. 2A,B). The preferential localisation of GBF1 to the ER-Golgi intermediate compartment (ERGIC) and cis-Golgi, with BIG1/2 localising to the trans-Golgi (García-Mata et al., 2003; Manolea et al., 2008; Yamaji et al., 2000) could support their differential regulation of Golgi compartments in non-adherent cells. The detection of GBF1 at the cis-medial Golgi in non-adherent cells (Fig. 5A) further helps this. GBF1 was reported to localise at focal adhesions in neuronal cells (Busby et al., 2017), though this was not detectable in fibroblasts. If confirmed, loss of adhesion-mediated disruption of focal adhesions could contribute to the recruitment of GBF1 at the cis-medial Golgi.

Arf1 possesses an N-terminal myristoylated amphipathic helix that anchors it to the Golgi membrane (Goldberg, 1998; Haun et al., 1993). GTP loading and activation of Arf1 triggers the release of the myristoylated N-terminal amphipathic helix, increasing its affinity for membranes. The Golgi-localised Arf-GEFs, while lacking a PH domain, carry multiple regulatory domains (Bui et al., 2009; Mouratou et al., 2005) required for their binding to Golgi membrane to regulate Arf1 activation (Bouvet et al., 2013; Christis and Munro, 2012; Gustafson and Fromme, 2017; Meissner et al., 2018; Pocognoni et al., 2018; Richardson et al., 2012). The interaction of GBF1 with Golgi membranes is vital for Arf1 activation at the cis-medial Golgi (Niu et al., 2005) and regulated by Rab1-mediated activation of PI4KIIIα and the resulting production of PtdIns(4)P on the Golgi membrane (Alvarez et al., 2003; Dumaresq-doiron et al., 2010; Monetta et al., 2007). Recent studies with Gea2, the yeast paralog of the human Arf-GEF GBF1, confirm the amphipathic helix to be specifically required for activating Arf1 on the membrane (Muccini et al., 2022). Active Arf1 at the Golgi is involved in the recruitment of COPI (Dascher and Balch, 1994), the Golgi-localized γ-ear-containing, Arf-binding proteins (GGAs), and adaptor protein (AP)-1 (Ooi et al., 1998; Traub et al., 1993). GBF1-mediated Arf1 activation supports the recruitment of COPI (Alvarez et al., 2003; Deng et al., 2009) and GGAs to Golgi membranes (Lefrançois and McCormick, 2007). Inhibition of GBF1 and the resulting loss of Arf1 activation leads to the dissociation of COPI driving the fragmentation of the Golgi and its fallback into the ER, inhibiting secretory traffic (Serafini et al., 1991; Tanigawa et al., 1993).

The fragmentation of the disorganised cis-medial Golgi in non-adherent fibroblasts by inhibition of Arf1 GEFs provides a unique cellular context for characterising GBF1-Arf1 mediated Golgi organisation. Increasing BFA and GCA concentration mediated inhibition of Arf1 GEFs and the resulting differences in the kinetics of decrease in active Arf1 levels drives differential cis-medial Golgi fragmentation (Figs 3 and 4). Increasing BFA/GCA concentrations do not significantly alter total Arf1 levels (Figs S1B, S1F, S2B, S2F), supporting a role for active Arf1 levels in mediating their effects on Golgi organisation. Over-expression of constitutive active Arf1 (Q71L), known to localise to the Golgi (Vasudevan et al., 1998), prevents BFA/GCA mediated Golgi fragmentation, further confirming this role (Fig. 5D,E). BFA and GCA treatment's comparable effects (Figs 3 and 4) support a role for GBF1-mediated regulation of Arf1 at the cis-medial Golgi (Fig. 5B,C). The detection of GBF1 at the cis-medial Golgi in suspended cells and its dispersal on Golgi fragmentation (Fig. 5A-C) confirms the same. GBF1-mediated regulation of the rate of Arf1 activation will affect the levels of active Arf1 associated with the Golgi. GBF1 and Arf1 are soluble proteins that rapidly cycle between their cytosolic and membrane-associated pools (Godi et al., 2004; Zhao et al., 2006). GBF1-mediated Arf1 activation and coatomer binding, combined with the recruitment of accessory proteins (McMahon and Mills, 2004; Orci et al., 1986; Reinhard et al., 2003), ultimately results in COPI vesicle formation. GBF1 and Arf1 membrane dynamics could significantly affect COPI assembly and disassembly (Bannykh et al., 2005; Kaczmarek et al., 2017; McMahon and Mills, 2004; Yu et al., 2012). Interestingly two GTP-bound Arf1 molecules bind sequentially to recruit the COPI heptameric coatomer (Dodonova et al., 2017; Yu et al., 2012). This suggests the complete disassembly of COPI coatomer would require the inhibition of both associated Arf1 molecules, which its kinetics of activation could influence. A rapid decrease in active Arf1 levels could hence allow COPI disassembly to be significantly enhanced, driving ER fallback. A more gradual reduction in active Arf1 levels could allow the known cycling of GBF1 and active Arf1 to rapidly restore their Golgi membrane levels to prevent complete COPI disassembly and Golgi fragmentation.

On loss of adhesion, the gradual drop (over 120 min) in active Arf1 levels to ∼50% of levels seen in stable adherent (SA) cells (Fig. 6A) supports Golgi disorganisation (but not fragmentation), which in turn regulates Golgi function (Singh et al., 2018). BFA and GCA treatment of non-adherent fibroblasts, rapidly decreasing active Arf1 levels to ∼50% of levels seen in SA cells in 10 min, causes the Golgi to fragment, disrupting its function (Fig. 6B-F). This suggests that the kinetics Arf1 inactivation (by regulating GEF) has vital implications for Golgi organisation in cells. While on the loss of adhesion this regulation causes a drop in Arf1 activation, a similar role for the kinetics of increase in Arf1 activation to restore Golgi organisation could also exist. Along with Arf GEFs, Arf GAPs could also contribute to the observed kinetics of change in active Arf1 levels to affect Golgi organisation and function. There could be a role for BFA-mediated regulation of ADP-ribosylation of cytosolic proteins like BARS in driving Golgi fragmentation (Colanzi et al., 2013; Corda et al., 2006; Mironov et al., 1997). However, this role is limited because GCA, which is not known to regulate ADP-ribosylation, similarly affects Arf1 inactivation kinetics and Golgi fragmentation in this study.

Along with loss of adhesion and possibly re-adhesion, such regulation of Arf1 and the Golgi could have implications for processes like cell division. A rapid change in cell adhesion accompanies it, and mitotic cell rounding (Dao et al., 2009; Dix et al., 2018) was accompanied by Golgi fragmentation and vesiculation, leading to its fallback into the mitotic ER (Colanzi and Corda, 2007; Corda et al., 2012; Shorter and Warren, 2002). It is mediated by AMPKα, Cdk1 and Casein kinase-2 mediated phosphorylation of GBF1, which regulates Arf1 activation (Magliozzi et al., 2018; Morohashi et al., 2010; Shaul and Seger, 2006) and possibly its kinetics. The dynamic nature of the Golgi organisation and its impact on cellular function makes its regulation very important to cells. The kinetics of Arf1 inactivation or activation in controlling Golgi organisation will provide cells with an additional means to fine tune Golgi and cell function.

## MATERIALS AND METHODS

### Reagents

FN (catalogue number F2006), BFA (B7651), and GCA (G0923) were purchased from Sigma. Phalloidin Alexa Fluor 488 (A12379) (Buwa et al., 2021; Joshi et al., 2012) was purchased from Invitrogen and Fluoromount-G (0100-01) was purchased from Southern Biotech. Concanavalin A Alexa-488 (ConA Alexa-488, C11252) was purchased from ThermoFisher Scientific. Antibodies used for western blots were: anti-Arf1 (clone 1D9, Abcam, ab2806) (Kulasekaran et al., 2021; Sadakata et al., 2010; Singh et al., 2018) at a dilution of 1:500 and anti-GAPDH (Abcam, G9545) (Lobato-Márquez et al., 2021; Singh et al., 2018) at a dilution of 1:5000, anti-GBF1 antibody at a dilution of 1:100 (Abcam, ab86071) (Jagdeo et al., 2018; Richards et al., 2014) used for immunostaining. Secondary antibodies conjugated to horseradish peroxidase (HRP) were purchased from Jackson Immuno Research and used at a dilution of 1:5000. Secondary antibody conjugated with Alexa-Flour 568 (A11036) was purchased from molecular probes. mCherry-tagged Arf1-WT and Arf1-Q71L constructs were made by releasing the Arf1 gene from GFP constructs (using BglII and BamH1 sites) and cloning the same into an empty mCherry-N1 vector. GalTase-RFP, Mannosidase II-GFP and ss-RFP-KDEL constructs were obtained from Dr Jennifer Lippincott-Schwartz (HHMI). GFP-ABD construct was provided by Professor Satyajit Mayor (NCBS).

### Cell culture, transfection, and suspension assay

Mouse embryonic fibroblasts (WT-MEFs) from Dr Richard Anderson (University of Texas Health Science Center, Dallas, TX) were cultured in complete Dulbecco's modified Eagle's medium (DMEM) (Invitrogen) with 5% fetal bovine serum (FBS) and penicillin-streptomycin (Pen-Strep; Invitrogen) at 37°C in a 5% CO_2_ incubator. Cell lines were routinely tested for Mycoplasma contamination and used only when clean. Cells were transfected using Lipofectamine 2000 (Invitrogen) in 6-cm dishes with a complete medium using 4 μg DNA for 12 h (for all constructs). Forty-eight h after transfection, cells were serum-deprived for 14 h in DMEM with 0.2% FBS and then used for experiments. Pathways regulated by cell-matrix adhesion are also known to be controlled by several growth factors in serum. Thus, cells were serum-deprived to clarify the effects of loss of cell-matrix adhesion. Serum-deprived cells were detached using trypsin-EDTA (Invitrogen) at 37°C and washed with low-serum DMEM. Cells suspended in 15 ml of low-serum DMEM were gently mixed with an equal volume of 2% methylcellulose in low-serum DMEM and incubated at 37°C for 120 min (120′ SUSP cells). Following this incubation, cells were collected at the required time, carefully washed twice with low-serum DMEM, and centrifuged at 1000 rpm for 5 min at 4°C. They were then reconstituted in low-serum DMEM and replated on coverslips coated with 2 μg/ml FN for 15 or 10 min (15′ FN or 10′ FN cells). Cells replated on FN were allowed to stay adherent for 4 h (stable adherent). For western blotting studies, suspended and re-adherent cells were lysed in 1× Laemmli buffer, heated at 95°C for 10 min, and stored at −80°C. For confocal microscopy, cells suspended or re-adherent on coverslips were washed twice with 1XPBS and fixed with 3.5% paraformaldehyde (PFA) for 15 min at room temperature. These cells were eventually washed with 1 x PBS, mounted using Fluoramount-G, and imaged using a Zeiss laser scanning microscope.

### Arf1 activity assay

To determine active Arf1 levels, 6×10^5^ cells (suspended or adherent) were washed following their respective incubation or drug treatment, frozen, and lysed in 400 µl activity assay buffer. Lysates were incubated with 60 µg of glutathione S-transferase (GST)-tagged Golgi localised γ-ear containing Arf-binding protein 3 (GGA3) fusion protein (GST-GGA3) (Pawar et al., 2016) to pull down active Arf1. 30 μl of the whole-cell lysate (6% of the total WCL) and all of the GGA3 pulldown sample (100% of total) was resolved by SDS-PAGE, western blotted using the anti-Arf1 antibody (Clone 1D9, Abcam). Blots were developed and imaged using the ImageQuant LAS 4000 (Fujifilm-GE); densitometric band analysis was done using ImageJ software (NIH). Arf1 band intensities from GGA3 pulldown were normalised to their respective whole-cell lysate. WT-MEFs treated with varying BFA and GCA concentrations for changing times were similarly processed, and their active Arf1 levels were calculated and normalised to their respective control samples.

### GFP-ABD mediated detection of active Arf1

Arf binding domain (ABD) encompasses a pleckstrin homology domain of ARHGAP10 that specially binds to active Arf1 (Kumari and Mayor, 2008). WT-MEFs were transfected with GFP-ABD (4 µg). Forty-eight h post-transfection, cells were serum-deprived for 14 h, detached and held in suspension for 120 min, replated on FN, and allowed to re-adhere for 10 min (early re-adhesion time point) and for 4 h (stable adherent time point). Cells were fixed with 3.5% PFA, immunostained with anti-GM130 antibody (1:100 dilution), mounted, and imaged using the confocal microscope. WT-MEFs were similarly transfected with GFP-ABD (4 µg) and GalTase-RFP (4 µg). Cells were suspended for 120 mins and replated on FN for 10 min (early re-adhesion) and 4 h (stable adherent). Fixed cells were then mounted and imaged using a Zeiss Confocal Microscope.

### RNA isolation and RT-PCR

RNA was isolated from WT-MEFs using Trizol reagent (Invitrogen), and cDNA was prepared using Oligo-dT primers and Reverse Transcriptase (Promega). Quantitative PCR was done using SYBR SAFE qPCR master mix in BioRad CFX96 Real-Time PCR System. The primers used are as follows: GBF1 forward: CGCACTCATAGATCCAACTC; GBF1 reverse: TCATCAGGACAACTTCATCAC; BIG1 forward: GCACATTGTCACTCTTGTATTT; BIG1 reverse: GTCGGATTGCTTCCATACTT; BIG2 forward: CTGCTAGGTTCTCTCACATTC; BIG2 reverse: TCG TGGGACTTTGGATCT.

### BFA or GCA treatment of suspended WT-MEFs for imaging

4×10^5^ WT-MEFs transiently transfected with ManII-GFP were serum-deprived for 14 h in low-serum DMEM (0.2% FBS), detached, and held in suspension for 60 min in DMEM with 1% methylcellulose. Cells were then treated with the required concentration of BFA or GCA and incubated for the required interval of time (10, 20, or 30 min, respectively) at 37°C. Control cells were treated with an equivalent volume of solvent (DMSO/methanol). Cells were processed as described above, and samples were collected at the required times.

### Determining the Golgi distribution profile

Cells expressing the cis-Golgi marker ManII-GFP or trans-Golgi marker GalTase-RFP were imaged using a confocal microscope. Golgi organisation was classified as organised, disorganised, partially fragmented, or fragmented based on the distribution of Golgi elements in the cells imaged by Confocal microscopy. Cis-medial marker ManII-GFP expression in cells was used to characterise Golgi organisation on inhibitor treatment. In cells with an intact/organised Golgi phenotype, the cis-medial ManII-GFP labelled Golgi has a distinct compact organisation. Cells with a disorganised Golgi were characterised by this compact organisation being marginally disrupted to cause the cis-medial ManII-GFP labelled Golgi to be more dispersed, keeping their perinuclear localisation in suspended cells. Partially fragmented cis-medial ManII-GFP labelled Golgi has a dispersed perinuclear Golgi pool but shows some diffused distribution throughout the cell. Occasionally GFP labelled tubular extensions form from the perinuclear pool in these cells. Man II-GFP-labelled Golgi partially overlap with the ssRFP-KDEL-labelled ER in these cells. The fragmented cis-medial ManII-GFP-labelled Golgi lack a perinuclear localisation and are distributed throughout the cell, appearing completely diffused in most cells. ManII-GFP-labelled tubular extensions are also seen throughout the cell. The fragmented Golgi were seen to overlap extensively with the ss-RFP-KDEL-labelled ER. Randomly selected cells (200 or more) in each population were evaluated for their Golgi organisation, as listed above. The percentage distribution of cells with each phenotype was calculated and plotted.

### Immunostaining for GBF1

WT-MEFs fixed with 3.5% PFA for 15 min at room temperature were permeabilised with 0.1% TritonX in 5% BSA for 30 mins at room temperature. They were blocked with 5% BSA in 1×PBS containing 10% horse serum overnight at 4°C. Coverslips were incubated with anti-GBF1 antibody (1:100 dilution) overnight at 4°C. Washed with 1×PBS and incubated with anti-mouse Alexa-568 (1:1000 dilution) at room temperature for 1 h. Cells were then mounted with fluoromount and imaged using a confocal microscope.

### Confocal microscopy and de-convolution of z-stacks

All imaging of cells was done using a Zeiss 710 or 780 laser scanning confocal microscope with a 63×oil objective (NA 1.4). Acquisition settings were: laser power=2%, pinhole=1 AU, and gain=650-900, and these settings were kept constant. Images were acquired at a resolution of 1024×1024, at a scan speed of 5. Z-stacks were acquired at 0.2 μm intervals and scan speed 7, de-convoluted using the Huygens Professional version 16.10 (Scientific Volume Imaging, the Netherlands, http://svi.nl). De-convoluted images were rendered as a maximum-intensity projection (MIP) using the MIP renderer plug-in.

### Colocalisation analysis

Colocalisation analysis was done using the Colocalization Analyzer plug-in in the SVI Huygens Professional software (version 16.10). Pearson correlation coefficients calculated were compared between treatments.

### Cell spreading quantitation

WT-MEFs re-adherent on FN for 15 min were stained with Phalloidin (1:500 dilution) and imaged using the EVOS FL Auto Imaging System (ThermoFisher Scientific, Waltham, MA, USA, AMAFD1000) at 40× magnification and analysed using the ImageJ software (NIH, Bethesda, MA, USA). First, phalloidin-stained cell images were processed, and a threshold was set to cover the entire stained cell. This was used to define the boundary of each cell using the tracing tool. Next, the total area within this mapped boundary for each cell was calculated using the “measure” option under the “analyse” tool in Image J. Finally, the area measured for 100 or more cells for each treatment or time point was used to calculate the mean cell spread area, which was then compared across treatments.

### Lectin labelling

WT-MEFs serum-deprived for 14 h were detached using Accutase, washed, and held in suspension for 10 min without or with BFA (3.6 µM) or GCA (0.5 µM). Post suspension, cells were harvested and incubated with ConA-Alexa 488 (0.05 µg/ml) for 15 mins on ice in the dark. Post incubation, cells were washed twice with cold 1×PBS and fixed with 3.5% paraformaldehyde for 15 mins, washed and resuspended in 1×PBS, and analysed using the instrument BD Celesta (BD Bioscience) and BD FACS Diva software. For each experiment, unlabelled WT-MEFs were used as a control to set voltage gates using FSC-A and SSC-A parameters. The gated population (P1) was selected to generate histograms for the FITC channel detecting the labelling of ConA Alexa488 lectin. The median fluorescent intensity values were obtained for comparing lectin labelling between samples.

### Multiple sequence alignment

The sequences of the mouse GEFs: BIG1(G3X9K3), BIG2 (A2A5R2), and GBF1(Q6DFZ1) were extracted from Uniprot. The Sec7 domains of these sequences were aligned along with the human ARNO sequence (Q99418) using Clustal Omega (www.ebi.ac.uk/Tools/msa/clustalo/) (DOI: 10.1038/msb.2011.75). Note that the Sec7 domains of the mouse GEFs were manually curated, keeping the human ARNO Sec7 domain from the structure of the ARNO-Arf1 complex (PDB ID:1R8Q) as a reference. The entire lengths of the four proteins were also aligned, not just their sec7 domains, as mentioned above. These results are not shown here and were not pursued further.

### Structure modelling of the mouse GEFs (BIG1, BIG2, GBF1) and mouse ARF1

The alphafold2-generated structures of the mouse BIG1, BIG2, GBF1, and Arf1 were taken from the EBI repository. These structures encompass the whole-length structures of all proteins. The sec7 domains of the GEFs, as defined for the alignment above, were extracted from these structures. To build a mouse Arf1GEF-Brefeldin-Arf1 complex, the MODELLER suite of programs (Šali and Blundell, 1993) was used for comparative modelling using the PDB structure of the human ARNO-BFA-Arf1 complex (PDB ID:1R8Q) as a template. The BFA-bound Arf1 undergoes a conformational change (well documented in Renault et al. 2003). The alphafold2 models did not incorporate these conformational changes and hence necessitated the use of MODELLER to construct models of the complex.

### Alpha fold models used for structural analysis

mouse GBF1: https://alphafold.ebi.ac.uk/entry/Q6DFZ1,

mouse BIG1: https://alphafold.ebi.ac.uk/entry/G3X9K3,

mouse BIG2: https://alphafold.ebi.ac.uk/entry/A2A5R2,

mouse Arf1: https://alphafold.ebi.ac.uk/entry/P84078.

### Statistical analysis

All the analysis was done using Prism GraphPad analysis software. Statistical analysis for Arf1 activity was done using a two-tailed unpaired Mann–Whitney test. Normalised data were analysed using the one-sample *t*-test.

## Supplementary Material

10.1242/biolopen.059669_sup1 Supplementary information
