# Supplementary material for: Kinetics of Arf1 inactivation regulates Golgi organisation and function in non-adherent fibroblasts
Source: Biol Open. 2023 May 4;12(4):bio059669. doi: 10.1242/bio.059669 (PMC10187640; doi:10.1242/bio.059669)
Supplement: Supplementary information [file biolopen-12-059669-s1.pdf]

Supplementary Figure 1

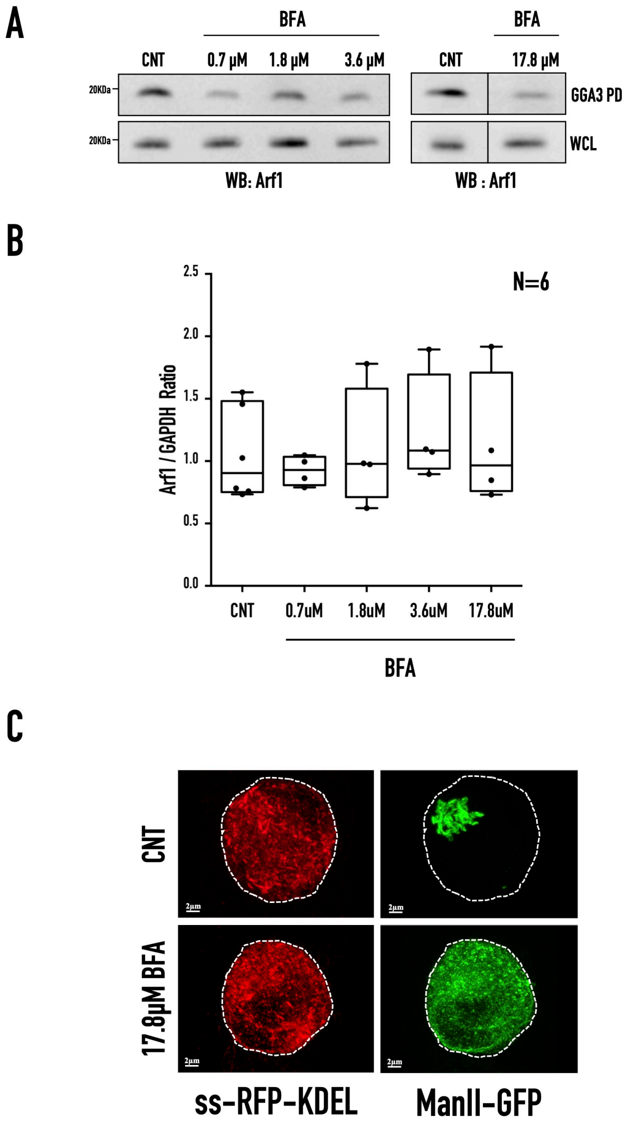

Biology Open • Supplementary information

Supplementary Figure 1

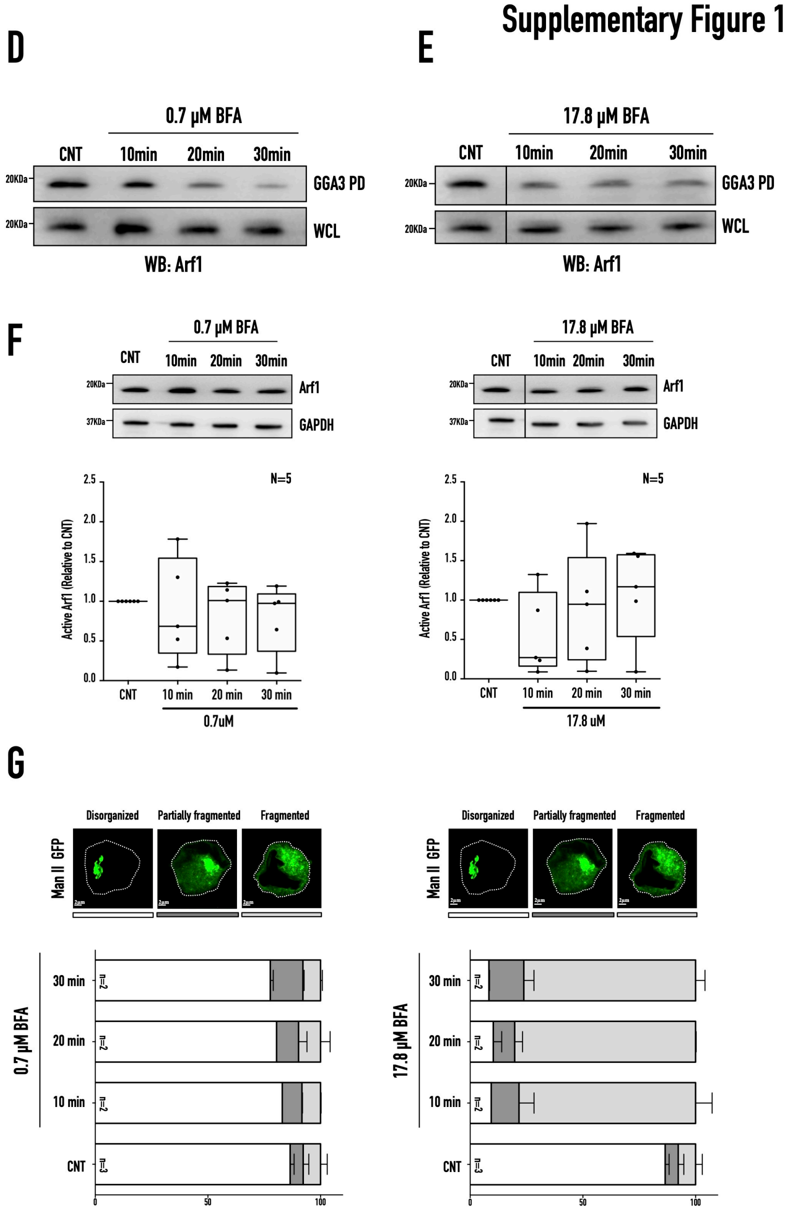

Biology Open • Supplementary information

Fig. S1. Concentration-dependent BFA mediated Arf1 inactivation in non-adherent WT-MEFs (A) Western blot detection of active Arf1 pulled down using GST-GGA3 beads (GGA3 PD) and total Arf1 levels in whole cell lysate (WCL) in WT-MEFs held in suspension for 60min and treated for 30min without (CNT) or with BFA 0.7µM, 1.8µM, 3.6µM and 17.8µM for 30min. 17.8µM BFA treated samples were run on a separate gel with the respective control sample. **(B)** Western blot detection of total Arf1 and GAPDH in WT-MEFs held in suspension for 60min and treated for 30min without or with increasing concentration of BFA (0.7µM, 1.8µM, 3.6µM and 17.8µM). The box and whisker plot represents the densitometric band intensity ratio of total Arf1 to GAPDH levels from 6 independent experiments. **(C)** Representative images of ss-RFP-KDEL (ER-marker) and ManII-GFP distribution in WT-MEFs suspended for 60min and treated without (CNT) or with 17.8 µM BFA for 30min (60' SUS+30min treatment. **(D, E)** WT-MEFs untreated (CNT) or treated with BFA 0.7µM **(D)** or 17.8µM **(E)**for 10min, 20min, and 30min, respectively. The gel image break shows the gap between the lanes where these samples were run. **(F)** Western blot detection of total Arf1 to GAPDH levels in WT-MEFs held in suspension for 60min and treated with 0.7µM or 17.8µM BFA for 10min, 20min and 30min, respectively. The gel image break shows the gap between the lanes where these samples were run. The box and whisker plots represent the western blot intensity ratio of Arf1 to GAPDH from 5 independent experiments. **(G)** Distribution profile depicting the percentage of cells with disorganised (white), partially fragmented (deep grey), and fragmented (grey) Golgi phenotype in non-adherent WT-MEFs, held in suspension for 60 min and treated without (CNT) or with 0.7µM or 17.8µM BFA for 10min, 20min and 30min, respectively. Representative images are shown for each Golgi phenotype described.

Biology Open • Supplementary information

Supplementary Figure 2

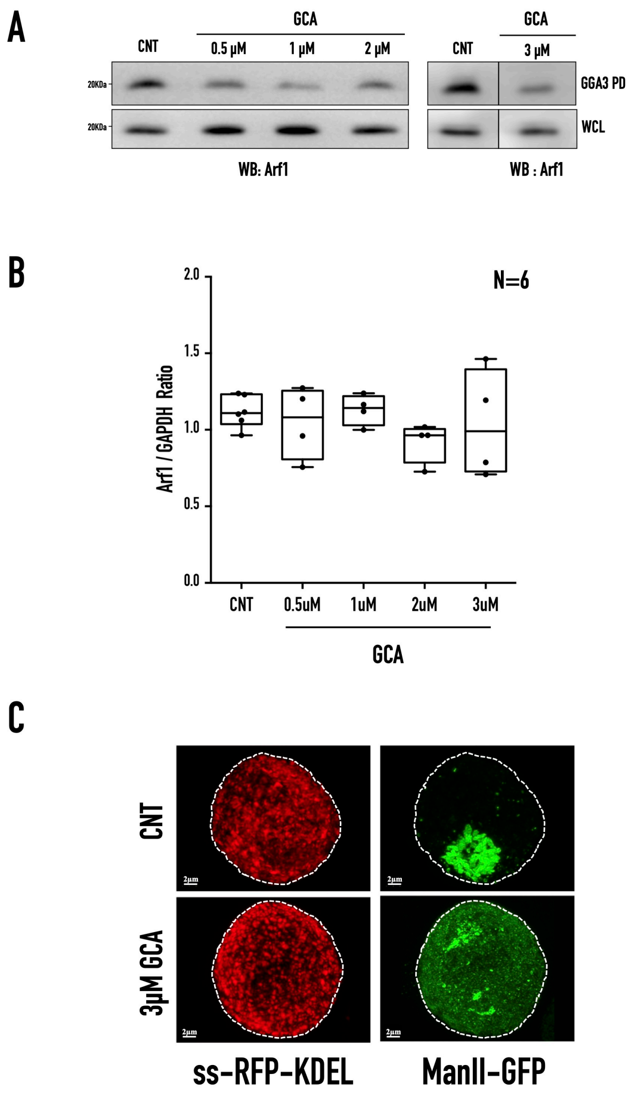

Biology Open • Supplementary information

Supplementary Figure 2

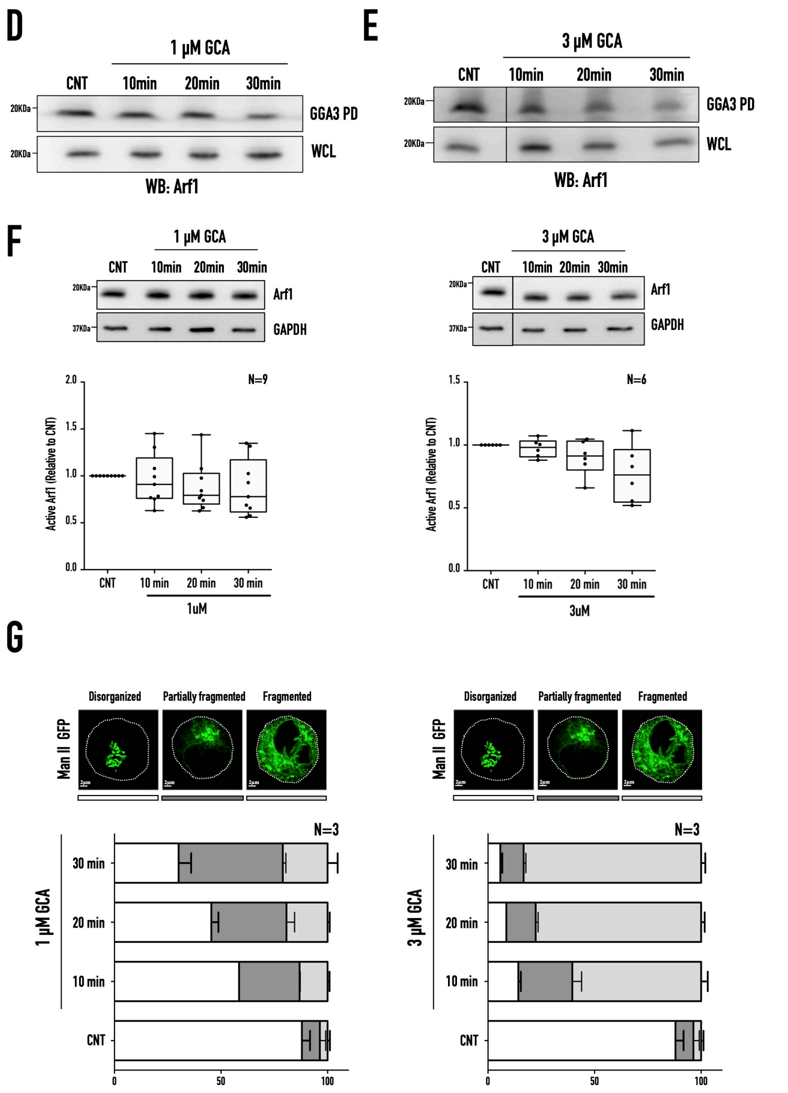

Biology Open • Supplementary information

Fig. S2. Concentration-dependent GCA mediated Arf1 inactivation in non-adherent WT-MEFs. (A) Western blot detection of active Arf1 pulled down using GST-GGA3 beads (GGA3 PD) and total Arf1 levels in whole cell lysate (WCL) in WT-MEFs held in suspension for 60 min and treated without (CNT) or with GCA 0.5µM, 1µM, 2µM and 3µM for 30 mins. 3µM GCA treated samples were run on a separate gel with the respective control sample. **(B)** Western blot detection of total Arf1 and GAPDH levels in WT-MEFs held in suspension for 60min and treated for 30min without or with increasing concentration of GCA (0.5µM, 1µM, 2µM, 3µM). The box and whisker plot represents the densitometric band intensity ratio of total Arf1 to GAPDH from 6 independent experiments. **(C)** Representative images of ss-RFP-KDEL (ER-marker) and ManII-GFP distribution in WT-MEFs suspended for 60min and treated without (CNT) or with 3µM GCA for 30min (60' SUS+30min treatment). **(D, E)** WT-MEFs untreated (CNT) or treated with GCA 1µM **(D)** or 3µM **(E)**for 10min, 20min, and 30min, respectively. The gel image break shows the gap between the lanes where these samples were run. **(F)** Western blot detection of total Arf1 to GAPDH levels in WT-MEFs held in suspension for 60min and treated with 1µM or 3µM GCA for 10min, 20min and 30min, respectively. The gel image break shows the gap between the lanes where these samples were run. The box and whisker plots represent the western blot intensity ratio of Arf1 to GAPDH from 9 or 6 independent experiments, respectively. **(G)** Distribution profile depicting the percentage of cells with disorganised (white), partially fragmented (deep grey), and fragmented (grey) Golgi phenotype in non-adherent WT-MEFs, held in suspension for 60 min and treated without (CNT) or with 1µM or 3µM GCA for 10min, 20min and 30min, respectively. Representative images are shown for each Golgi phenotype described.

Biology Open • Supplementary information

Supplementary Figure 3

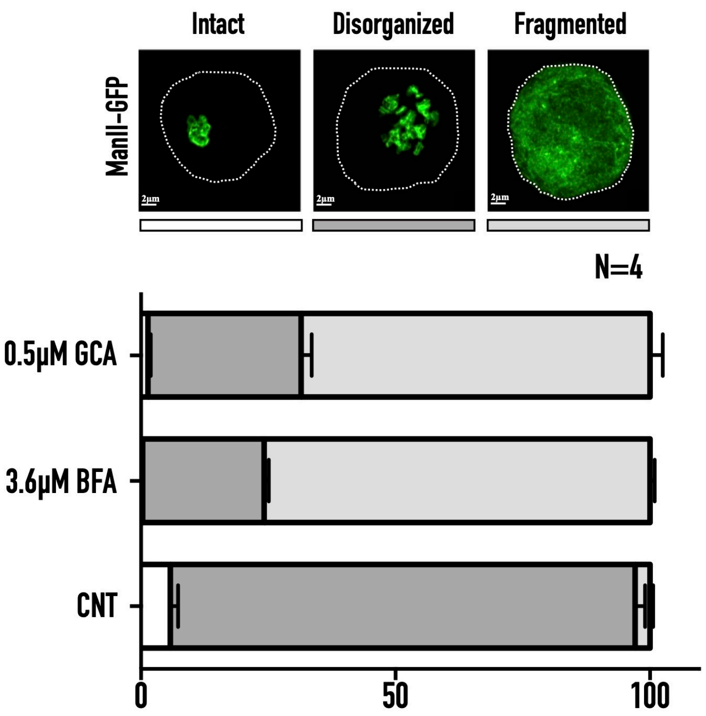

Fig. S3. Distribution profile represents the percentage of cells with intact (white), disorganised (dark grey), or fragmented (grey) Golgi phenotype in non-adherent WT-MEFs, untreated or treated with 3.6µM BFA or 0.5µM GCA for 10min in suspension. Representative images are shown for each Golgi phenotype described. The graph represents mean±SE from 4 independent experiments (at least 200 cells counted for each sample per experiment).

Biology Open • Supplementary information
